# NLR, MLP, SVM, and LDA: a comparative analysis on EMG data from people with trans-radial amputation

**DOI:** 10.1186/s12984-017-0290-6

**Published:** 2017-08-14

**Authors:** Alberto Dellacasa Bellingegni, Emanuele Gruppioni, Giorgio Colazzo, Angelo Davalli, Rinaldo Sacchetti, Eugenio Guglielmelli, Loredana Zollo

**Affiliations:** 1 Research Unit of Biomedical Robotics and Biomicrosystems, Università Campus Bio-Medico di Roma, v. Alvaro Del Portillo, Rome, Italy; 2 Centro Protesi INAIL di Vigorso di Budrio, v. Rabuina, Bologna, Italy

**Keywords:** Prostheses, Surface electromyography, Nonlinear logistic regression, Support vector machine, Multi-layer perceptron, Linear discriminant analysis, Pattern recognition, Statistical comparison, Embedded

## Abstract

**Background:**

Currently, the typically adopted hand prosthesis surface electromyography (sEMG) control strategies do not provide the users with a natural control feeling and do not exploit all the potential of commercially available multi-fingered hand prostheses. Pattern recognition and machine learning techniques applied to sEMG can be effective for a natural control based on the residual muscles contraction of amputated people corresponding to phantom limb movements. As the researches has reached an advanced grade accuracy, these algorithms have been proved and the embedding is necessary for the realization of prosthetic devices. The aim of this work is to provide engineering tools and indications on how to choose the most suitable classifier, and its specific internal settings for an embedded control of multigrip hand prostheses.

**Methods:**

By means of an innovative statistical analysis, we compare 4 different classifiers: Nonlinear Logistic Regression, Multi-Layer Perceptron, Support Vector Machine and Linear Discriminant Analysis, which was considered as ground truth. Experimental tests have been performed on sEMG data collected from 30 people with trans-radial amputation, in which the algorithms were evaluated for both performance and computational burden, then the statistical analysis has been based on the Wilcoxon Signed-Rank test and statistical significance was considered at *p* < 0.05.

**Results:**

The comparative analysis among NLR, MLP and SVM shows that, for either classification performance and for the number of classification parameters, SVM attains the highest values followed by MLP, and then by NLR. However, using as unique constraint to evaluate the maximum acceptable complexity of each classifier one of the typically available memory of a high performance microcontroller, the comparison pointed out that for people with trans-radial amputation the algorithm that produces the best compromise is NLR closely followed by MLP. This result was also confirmed by the comparison with LDA with time domain features, which provided not significant differences of performance and computational burden between NLR and LDA.

**Conclusions:**

The proposed analysis would provide innovative engineering tools and indications on how to choose the most suitable classifier based on the application and the desired results for prostheses control.

## Background

In clinics the state-of-the-art technology for people with trans-radial amputation is commonly a dual-site controlled myoelectric hand prosthesis. The available single degree of freedom is actuated by applying a simple threshold or a proportional amplitude method on surface electromyography (sEMG) signals recorded from antagonistic muscles (e.g., wrist flexor and wrist extensor) that can be easily contracted in a separate way. In the case of multi-fingered hand prosthesis with several degrees of freedom (DoFs), but still having two control signals, the switching between DoFs or predefined grasps is normally made by co-contraction, as in a finite state machine. This serial operation is slow and unnatural; in addition, it requires considerable training and cognitive effort [1].

On the other hand, Targeted Muscled Re-innervation (TMR) [2], via surgical operation, allows replacing nerves from the stump of persons with amputation to different anatomical muscles (e.g., chest muscles) in order to obtain independent signals. The risk associated to the surgical re-innerving operation is the main drawback that limits the applicability of this technique to all the kinds of amputations [3, 4].

Pattern recognition techniques based on sEMG currently represent the best compromise between invasiveness and prosthesis controllability and thanks to the notable scientific progress, allows increasing the number of controllable DoFs by keeping low the number of utilized electrodes [5]. Recognizing the user’s will, control strategy resorting to pattern recognition techniques could improve performance by mapping the actuation of the prostheses on sEMG signals produced as result of phantom limb gestures [6]. The system becomes more user-friendly, and makes easier complex tasks that may include the sequential actuation of different DoFs.

**Fig. 1:**
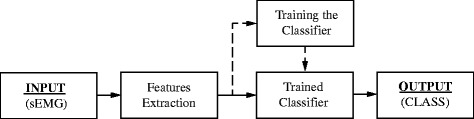
Block diagram of a generic pattern recognition system based on sEMG signals

Myoelectric control systems based on pattern recognition techniques (Fig. 1) rely on supervised machine learning classification algorithms.

An initial training phase is needed, during which the system learns the way of linking the gestures to specific myoelectric patterns. Subsequently, the trained system is able to find out, from recorded patterns, the function for realizing and executing the desired task. Usually the feature extraction step precedes classification of sEMG signals where the most important components of the recorded myoelectric signal on a chosen time window are identified and selected [7] in order to improve the stability of the features (reducing variance and increasing classification performance). Previous studies suggest that the optimum window length for pattern recognition controls ranges from 150 to 250 ms depending on the skill of the subject [8]. For real-time applications it is conventionally accepted that the actuation delay must be less than 300 ms, therefore it was proposed to use a method for adopting “raw” filtered sEMG signals as input features, which enables an extreme reduction of the classification time and of the response time of the system without significant loss of system performance [9–11]. The saved time is used to improve the stability of the classification by means of post processing techniques as voting and/or threshold policies [12, 13].

Linear classifiers, such as Linear Discriminant Analysis (LDA), Logistic Regression (LR) or Support Vector Machine (SVM) with linear kernel, and nonlinear classifiers, such as Non-linear Logistic Regression (NLR), SVM with nonlinear kernels and Multi-Layer Perceptron (MLP), represent the state-of-the-art about pattern recognition classifiers [14, 15]. The main difference between linear and nonlinear classifiers consists in the shape of the decision boundary: straight line, or plane in the first case and curved line, or surface, in the second. Performance, complexity and computational time usually increase together. Hence, the choice of a classification algorithm should not be entirely relied upon performance, but rather on a trade-off between computational burden and performance, especially in embedded systems. This work aims to provide useful insights into the choice of the suitable classifier (and its specific internal settings) for the embedded control of multi-fingered hand prostheses. To this purpose, a comparative analysis among NLR, MLP, SVM with Radial Basis Function (RBF) kernel, and LDA with time domain feature extraction, considered as benchmark classifier, on sEMG data from 30 people with trans-radial amputation is carried out, in terms of performance and computational burden. The use of LDA with time domain feature extraction in on-line control of prosthetic devices has been demonstrated by several studies [16, 17]; this method is now commercially available in the US by COAPT https://www.coaptengineering.com.

This paper is structured as follows: Sect. II describes the protocol for the acquisition of the sEMG datasets, the implemented machine learning algorithms, and the methods adopted for data analysis; Sect. III reports the results of a preliminary analysis on the complexity range of the model of NLR and MLP, then it reports the comparative analysis among the NLR, MLP and SVM classifiers including a combined index of performance and computational burden for the evaluation of the most suitable classifier for the embedding version on a microcontroller with a 256 KB of memory for the realization of a prosthetic device. The section concludes with a comparative analysis between NLR and the ground truth represented by LDA with time domain feature extraction. Conclusive remarks are finally reported in Sect. V and VI.

## Methods

### sEMG data acquisition protocol

**Fig. 2:**
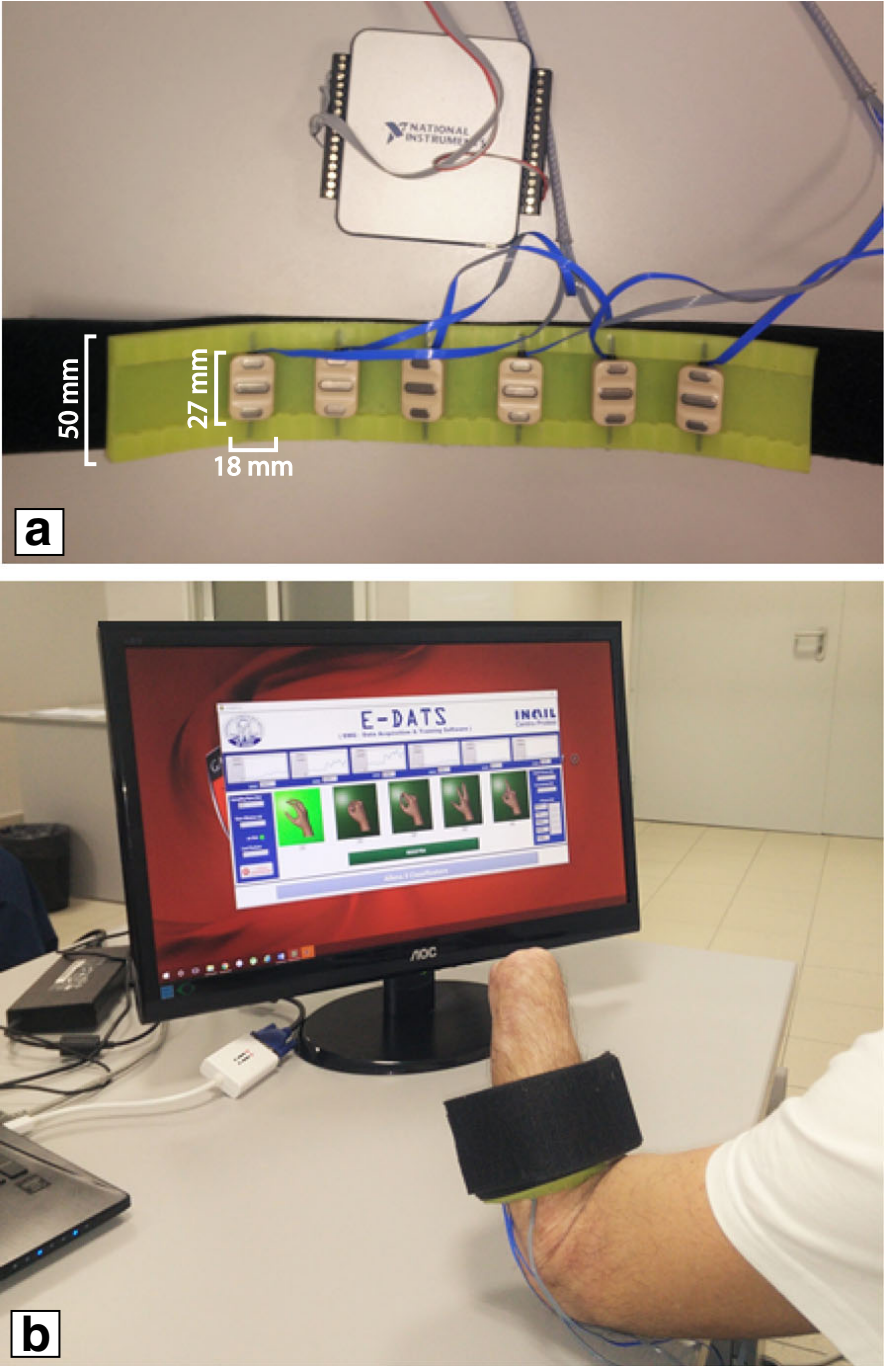
Experimental Setup **a**) sEMG bracelet and NI DAQ USB 6002; **b**) Subject positioning and acquisition Software

The same acquisition protocol as in [18] was used to collect the sEMG data from the subjects participating in the experiments. Thirty people with trans-radial amputation, aged between 18 and 65, free of known muscular and/or neurological diseases, participated in the experiments. Each subject gave informed consent before performing the experiments, which were approved by local scientific and ethical committees, and were already experienced in myoelectric control of prosthetic hands. Six commercial active sEMG sensors (Ottobock 13E200 = 50, 27 mm × 18 mm × 9.5 mm) were equidistantly placed on a silicone adjustable bracelet (Fig. 2a) and were fastened on subject’s stump (Fig. 2b). These sensors operate in the range 0–5 V with a bandwidth of 90–450 Hz and a common rejection ratio higher than 100 dB. The first sensor was located on the flexor carpi-radialis muscle, while the sixth sensor on the brachio-radialis muscle. These two muscles were identified by manual inspection of the stump; then, sEMG sensors were equally spaced each other on the silicone bracelet. The bracelet was located about 5 cm below the subject’s elbow, in line with the positioning of the electrodes, commonly used to control the myoelectric prosthesis. The data was collected using a purpose built software on LabView platform by means of a NI DAQ USB 6002 device in order to sample the six sEMG signals at 1 kHz frequency and with 12 bits resolution.

**Fig. 3:**
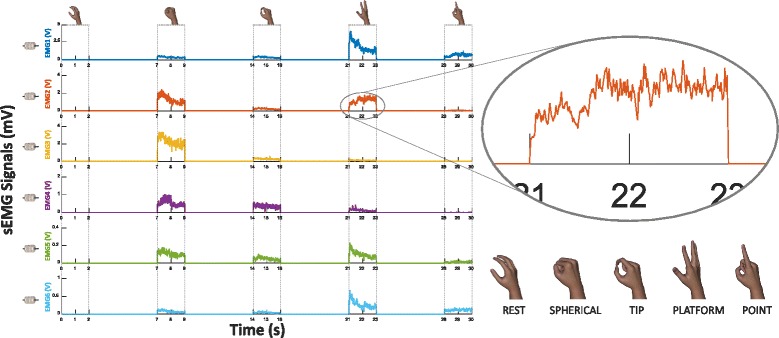
Graphic display of the selected gestures and of the raw recording for the six different channels at the same time for all the imagined movements of a single acquisition session from one of the subjects who took part to the experiment

Each subject was sitting in a comfortable chair in front of a PC monitor (Fig. 2b), where one of five hand gestures was randomly shown. The subjects were instructed to reproduce steady state the displayed gesture with their phantom limb. Once the signals became stable the sampling session started and continued for 2 s obtaining for each sensor 2000 samples. The gestures to reproduce were selected among the eight canonical hand postures [7, 19] and were “*Rest”* (relaxed hand), “*Spherical”* (hand with all fingers closed), “*Tip”* (hand with thumb and finger touching to pick up a small object), “*Platform”* (hand completely open and stretched), and “*Point”* (hand with all fingers closed, except for the index finger that is pointing). Each acquisition started from *“Rest”* position; after two seconds of acquisition, the subjects were asked to return to the *Rest* posture. Moreover, the subjects were instructed to accomplish the task with the minimum muscular contraction and focus on the main phantom fingers related to the gesture. The selected gesture was shown as in Fig. 3. Ten repetitions of each gesture were accomplished in a single acquisition session with an inter-stimulus interval of about 5 s. Figure 3 also shows a case of the raw recording from the six sEMG sensors for all the imagined movements. The plot is related to a single acquisition session from one of the subjects who took part to the experiment.

### NLR, MLP, and SVM classification algorithms

In order to obtain a fast response real-time classification no feature extraction was performed from the recorded signals, hence the sEMG signal are used directly as input for the classification algorithms. The unique operation done on sEMG signals is the scaling. It consists of subtracting the mean value to each signal and dividing the result by the range. Hence, for each time step (*i*) we obtain a six-element vector *x* ^(*i*)^ of scaled sEMG signals, which is used as input for the classifiers to compare, i.e.: NLR, MLP, LDA, and SVM with RBF kernel. Supervised machine learning techniques are commonly adopted in problems where there is no functional relationship *y* = *f*(*x*) that binds the inputs *x* ^(*i*)^ with the corresponding class (*y*). There are two different approaches to classification: the first one returns a distribution *P*(*y*| *x*); the second one returns a result without any probability of class membership [20].

LR [21], or Perceptron, is a linear and binary supervised classification algorithm that calculates the class membership, probability using the following logistic function

1 $$ P\left(1|x,\theta \right)=\left\{\begin{array}{l}g\left({\theta}^T\cdot x\right)=\frac{1}{1+{e}^{-\left({\theta}^T\cdot x+{\theta}_0\right)}}\hfill \\ {}1-P\left(y=0|x,\theta \right)\kern2.04em ,\hfill \end{array}\right. $$

where *θ* and *θ*_0_ are the classification parameters vector and the bias term, respectively, and *g*(∙) is the logistic, or sigmoid, function. In order to achieve a NLR the creation of additional input features (*interaction terms*) is needed. For this study, additional polynomial features were used, which were obtained as a combination product of the starting input features (e.g., *x*_1_ ; *x*_2_ ; *x*_1_ · *x*_2_ ; *x*_1_^2^ ; *x*_2_^2^ ; …). The prediction of class labels (*h*_*θ*_) for LR or NLR algorithm is then achieved by comparing the distribution *P*(*y*| *x*) with a *decision threshold* (*TH*) as

2 $$ {h}_{\theta }(x)=\left\{\begin{array}{l}P\left(1|x,\theta \right)\ge TH\to 1\hfill \\ {}P\left(1|x,\theta \right)< TH\to 0.\hfill \end{array}\right. $$

MLP [20, 21] is a particular case of supervised Artificial Neural Network (ANN) where each node, or neuron, of the architecture implements a logistic function. The network architecture has an input layer, one or more hidden layers (with the same number of neurons), and an output layer with one neuron for each class to be classified. The output vector of the *l-th* layer (*a* ^(*l*)^) of this particular classifier is obtained through *forward propagation* as

3 $$ {a}^{(l)}=\left\{\begin{array}{l}x,\hfill \\ {}g\left({\varTheta}^{\left(l-1\right)}\cdot {a}^{\left(l-1\right)}+{\varTheta}_0^{\left(l-1\right)}\right)\kern0.62em ,\hfill \end{array}\right.\kern2.1em {\displaystyle \begin{array}{l}l=1.\hfill \\ {}l=2,\kern0.5em 3,\kern0.5em \dots, \kern0.5em L.\hfill \end{array}} $$

Where Θ^(*l*)^, Θ_0_^(*l*)^ are the classification parameters matrix and the bias vector associated with the *l-th* layer, respectively, and *L* indicates the output layer. Hence, the output of the network is a vector *Pv*(*y*| *x*) whose elements represent the class membership probability expressed as

4 $$ Pv\left(y|x,{\varTheta}^{(l)},{\varTheta}_0^{(l)}\right)={a}^{(L)}.l=1,\kern0.5em 2,\dots, \kern0.5em L. $$

Also for MLP it is possible to achieve the prediction of class labels (*h*_Θ_) by comparing each value of the distribution vector *Pv*(*y*| *x*) with *TH* and assigning to *h*_Θ_ the index of the element of *Pv*(*y*| *x*) that represents the maximum among all those resulted above the decision threshold.

SVM [20, 21] is a linear and binary supervised classification algorithm that considers only dichotomous distinction between two classes, and assigns class label 0 or 1 to unknown data item [20] as follows

5 $$ {h}_{\theta }(x)=\left\{\begin{array}{l}\left({\theta}^T\cdot x+{\theta}_0\right)\ge +1\to 1\hfill \\ {}\left({\theta}^T\cdot x+{\theta}_0\right)\le -1\to 0\kern0.36em .\hfill \end{array}\right. $$

In order to obtain a nonlinear classifier, a *kernel function* needs to be included into the model. A kernel function is a similarity function (*f*), satisfying the Mercer’s Theorem, that expresses the similarity between the generic input vector *x* and a *landmark* (*s*), representing one of the the two classes. Typically a selection of all the *x* vectors recorded for training the SVM algorithm are set as landmarks and the *j-th* element of *f* for a RBF kernel becomes

6 $$ {f}_j=\exp \left[-\frac{{\left|x-{s}^{(j)}\right|}^2}{2\gamma}\right],\kern2em j=1,\kern0.5em 2,\kern0.5em \dots, \kern0.5em n. $$

where *n* is the number of landmarks chosen as representative vector of classes 0 and 1, and *γ* is the internal RBF parameter. Then the input features vector becomes *f* and the class labels for a SVM with RBF kernel^1^ are assigned as

7 $$ {h}_{\theta }(f)=\left\{\begin{array}{l}\left({\theta}^T\cdot f+{\theta}_0\right)\ge +1\to 1\hfill \\ {}\left({\theta}^T\cdot f+{\theta}_0\right)\le -1\to 0.\hfill \end{array}\right. $$

Classification parameters *θ*, *θ*_0_, Θ^(*l*)^, and Θ_0_^(*l*)^ are obtained from the minimization of a particular cost function *J*(∙) associated with each classifier,

### NLR, MLP, SVM classifiers and optimization algorithm implementation

NLR, MLP and SVM classification algorithms were implemented in MATLAB. For NLR and MLP the code was ad-hoc developed, while for SVM the open source library libsm3.20 [22] was used. The developed function that implements NLR allows the user to choose the maximum value of the variable D, which encodes a structure of polynomial features as reported in Table 1.

**Table 1:** Encoding the variable D

D	Description	Example
1	Linear case (LR)	x_1_, x_2_, x_3_, x_4_, x_5_, x_6_
2	max 2nd degree	x_1_, …, x_6_, x_1_x_2_, x_1_x_3_, …, x_5_x_6_, x_1_^2^, x_2_^2^, …, x_6_^2^
3	max 3rd degree	x_1_, …, x_6_^2^, x_1_x_2_x_3_, …, x_4_x_5_x_6_, x_1_^3^, …, x_6_^3^
4	max 4th degree	x_1_, …, x_6_^3^, x_1_x_2_x_3_x_4_, …, x_3_x_4_x_5_x_6_, x_1_^4^, …, x_6_^4^
5	max 5th degree	x_1_, …, x_6_^4^, x_1_x_2_x_3_x_4_x_5_, …, x_2_x_3_x_4_x_5_x_6_, x_1_^5^, …, x_6_^5^
6	max 6th degree	x_1_, …, x_6_^5^, x_1_x_2_x_3_x_4_x_5_x_6_, x_1_^6^, …, x_6_^6^
7	max 7th degree	x_1_, …, x_6_^6^, x_1_^7^, x_2_^7^, x_3_^7^, x_4_^7^, x_5_^7^, x_6_^7^

As polynomial features are intended the starting features high till the indicated degree and all the multiplications that arise from the possible permutations without repetitions of a maximum number of elements corresponding to the indicated degree. A *cross-entropy error* cost function has been associated to the NLR algorithm and is expressed a

8 $$ J\left(\theta, {\theta}_0\right)=-\frac{1}{m}\left[\sum \limits_{i=1}^m{y}^{(i)}\cdot \ln g\left({\theta}^T\cdot {x}^{(i)}+{\theta}_0\right)\right]-\frac{1}{m}\left[\sum \limits_{i=1}^m\left(1-{y}^{(i)}\right)\cdot \ln \left(1-g\left({\theta}^T\cdot {x}^{(i)}+{\theta}_0\right)\right)\right], $$

where *m* is the number of samples used to train the algorithm and *y* ^(*i*)^ is the known class membership of the *i-th* sample. Being NLR a binary classification algorithm, a *one* vs. *all* approach was implemented to address the multi-class classification problem.

The developed function that implements MLP allows the user to decide the maximum number of hidden layers and the maximum number of neurons for each of them. A *mean square error* cost function has been associated to the MLP algorithm, as

9 $$ J\left(\varTheta, {\varTheta}_0\right)=\frac{1}{m}\sum \limits_{i=1}^m\sum \limits_{k=1}^K{\left[{y}_k^{(i)}-{\left({a}_k^{(L)}\right)}^{(i)}\right]}^2, $$

where *K* is the number of classes to be recognized, ${y}_k^{(i)}$ is the known *k-th* element of the class membership vector of the *i-th* sample, and ${a}_k^{(i)}$ is the *k-th* element of the evaluated membership probability vector of the *i-th* sample.

As previously mentioned, the SVM classifier with RBF kernel has been developed exploiting the open source library *libsvm3.20* that is widely used for multiclass machine learning problems. More detailed information can be found in [22–24]. Anyway the cost function *J*(∙) associated to the SVM algorithm can be expressed as

10 $$ J\left(\theta, {\theta}_0\right)=-C\left[\sum \limits_{i=1}^m{y}^{(i)}\cdot \ln g\left({\theta}^T\cdot f+{\theta}_0\right)\right]-C\left[\sum \limits_{i=1}^m{y}^{(i)}\cdot \ln \left(1-g\left({\theta}^T\cdot {x}^{(i)}-{\theta}_0\right)\right)\right]+\frac{1}{2}\left[{\theta}^T\cdot \theta +{\left({\theta}_0\right)}^2\right], $$

The developed function allows the user to set the value regularization parameters C that appear into the cost function implemented in libsvm3.20 and the value of the internal RBF parameter γ. In this case, to address the multiclass classification problem it has been chosen to rely on a *one* vs. *one* method as recommended by the developers for practical usage of the library [23–30]. 

Since each of the aforementioned classifiers requires to set internal parameters, in addition to classification parameters *θ*, *θ*_0_, Θ^(*l*)^, and Θ_0_^(*l*)^, it is coupled with an iterative optimization algorithm. The optimization strategy relies on a *three ways data split approach* [25]. Hence, the initial data set is divided into three subsets: *“Training Set”* (TR) containing 60% of the data, *“Cross Validation Set”* (CV) containing 20% of the data, and *“Test Set”* (TS) containing the remaining 20% of the data. These subsets are iteratively filled through a *random shuffle* until a configuration with a proportionated class number is reached. The TR is used to train the supervised classification algorithms by minimizing the specific cost function. As minimization algorithm, Resilient Backpropagation (RProp) [26] has been chosen for NLR and MLP and Limited memory Broyden-Fletcher-Goldfarb-Shanno (L-BFGS) [27] for SVM. Each single classifier is iteratively trained with all the possible configurations of its internal parameters, varying each of these within an appropriate range of values. The CV is then used to evaluate performance of each configuration (i.e. model), in order to avoid overfitting and find out the best model.

The F1Score [28] was used in this study to assess performance, in lieu of accuracy, being more robust also for classes that do not have a perfect symmetrical cardinality. Considering this simple confusion matrix


11


Where *nP* is the number of true positive, *nN* the number of true negative, *nFP* the number of false positive and *nFN* the number of false negative, F1Score can be evaluated as

12 $$ \left\{\begin{array}{c}\hfill PR=\frac{nP}{\left( nP+ nFN\right)}\kern2.759999em \hfill \\ {}\hfill RE=\frac{nP}{nP+ nFP}\kern3.239999em \hfill \\ {}\hfill F1 Score=2\cdot \frac{PR\cdot RE}{PR+ RE}\cdot 100,\hfill \end{array}\right. $$

where *PR* is called *Precision* and *RE* is called *Recall*.

After determining the optimal classifier model, the TS is used to achieve an estimation of the performance that the classifier is expected to show when new features are provided as input.

### NLR, MLP, SVM Downsampling and creation of generalization set

For each subject involved in the experiment, the data sampled at 1 kHz were organized in a matrix; each column of the matrix was coupled with an EMG sensor. Hence, the choice of avoiding features extraction based on time windowing of sEMG generated 10^5^ × 6 data (large-scale datasets) and, consequently, a very long time (more than 4 h per subject) is required to complete training and optimization for each classification algorithm. Therefore, downsampling has been applied to speed up the whole process. The discarded data were used to compose a new set of data called *“Generalization Set”* (GS) which has been used as second test set in order to obtain an estimation of the generalization ability of each classification algorithm. In particular, for a downsampling step equals to 10 (one in ten), the GS will contain 90% of the data, the TR 6%, the CV 2%, and the TS the remaining 2% of the data. In other terms, the results evaluated on TS represent an estimation of the classification ability when the signal to classify is sampled at the same frequency of the training data (a downsampling step equals to 10 produce a 100 Hz dataset) while results evaluated on GS represents an estimation of the classification ability when classifying a signal sampled up to 1 kHz.

### LDA classifier

LDA is a linear and binary supervised classification algorithm that considers a dichotomous distinction between two classes, and assigns class label 1 or 2 to unknown data item relying on the following decision function

13 $$ {h}_{\beta }(x)=\left\{\begin{array}{l}\left({\beta}^T\cdot x+{\beta}_0\right)\ge 0\to 1\hfill \\ {}\left({\beta}^T\cdot x+{\beta}_0\right)<0\to 2\kern0.36em ,\hfill \end{array}\right. $$

where *β* and *β*_0_ are the classification parameters vector and the bias term, respectively. Classification parameters can be evaluated as

14 $$ \left\{\begin{array}{l}\beta ={\varSigma}^{-1}\cdot \left({\mu}_1-{\mu}_2\right)\hfill \\ {}{\beta}_0=-{\beta}^T\cdot \left(\frac{\mu_1+{\mu}_2}{2}\right)+\ln \left(\frac{\Pi_1}{\Pi_2}\right)\kern0.36em ,\hfill \end{array}\right. $$

where Σ is the pooled covariance matrix, *μ*_1_, *μ*_2_ and Π_1_, Π_2_ are the mean vectors and the prior probabilities of class 1 and class 2, respectively. Since this classifier does not require setting internal parameters, training and test rely on a *two ways data split approach* [25]. Hence, the initial dataset is divided into training set and test set. The training set contains 70% of the data (TR_70%_), and the test set contains the remaining 30% of the data (TS_30%_). The subsets are iteratively filled through a *random shuffle* until a configuration with proportionated class number is reached. The TR_70%_ is used to train the classifier evaluating classification parameters *β* and *β*_0_; on the other hand, the TS_30%_ is used to estimate the classifier performance when new features are provided as input. Being LDA a binary classification algorithm, a *one* vs. *all* approach was implemented to address the multi-class classification problem. The class label (*c*) is predicted as

15 $$ {h}_{\beta }(x)=\underset{c}{\max \limits}\left({{}_c\beta}^T\cdot x+{{}_c\beta}_0\right)\mathrm{and}\kern2.04em \left\{\begin{array}{c}\hfill {}_c\beta ={\varSigma}^{-1}\cdot {\mu}_{c\kern8.519994em }\kern0.24em \hfill \\ {}\hfill {{}_c\beta}_0=-{{}_c\beta}^T\cdot \left(\frac{\mu_c}{2}\right)+\ln \left({\Pi}_c\right),\hfill \end{array}\right. $$

where ${}_c{}\beta$ and ${{}_c{}\beta}_0$ are the classification parameters vector and the bias term of *c* class, respectively. For building our LDA benchmark classifier five commonly used time domain features were considered^2^: Mean Absolute Value (MAV), Root Mean Square (RMS), Slope Sign Change (SSC), Waveform Length (WL) and Variance (*σ* ^2^). They were extracted in windows of 250 ms with an overlap of 200 ms [17]. Since the training of the LDA classifier is performed by means of Eq. (13, 14) and the feature extraction avoids the generation of large-scale-dataset, a short time is required to complete the training of the classifier and there is no need to perform down sampling. The classification algorithm was implemented in MATLAB with an ad hoc developed software code.

### Data analysis

The study was divided into three parts: the first one investigated the optimal range of D (initial guess 1–7) for NLR, and the range of maximum number of layers (initial guess 1–10) and neurons (initial guess 1–30) for MLP, while the second part is focused on the comparison among the NLR, MLP and SVM classification algorithms. The third part is focused on the comparison with our ground truth, the LDA classifier. The first part can be seen as a preliminary investigation in order to reduce the evaluation time of the comparison among the three classifiers. A downsampling step equal to 10 (and corresponding to a 100 Hz sampling frequency) has been applied to data collected from 30 people with trans-radial amputation. Performance of each algorithm has been measured by means of the F1Score (12) value and a statistical analysis has been based on the Wilcoxon Signed-Rank test, which has been shown to be appropriate for comparing different classifiers in common datasets [1-29]. Statistical significance was considered at p < 0.05. The maximum value of D, of the number of layers, and of the number of neurons have been obtained by means of a sequential statistical analysis, starting from the simplest case and then sequentially comparing all the others until a high significant difference of performance is found. This is taken as the new benchmark for all the subsequent comparisons. The process ends when it is found the last case in which the differences are not statistically significant compared to all subsequent cases.

The second part, the core of our work, resorted to the results obtained in the first part to compare NLR, MLP, and SVM considering both performance and run-time computational burden on EMG data collected from 30 people with trans-radial amputation. As regards the SVM, the range of variation of the regularization parameter C belongs to 0–10^4^, with variable steps starting from 0.01 and doubling each time, while γ belongs to 0–50 (with a pitch equals to 0.1); both have been empirically determined in previous tests. The computational burden was evaluated through the number of parameters (nθ), expressing the cardinality of classification vector θ (1) (7) or matrices Θ (3) that identify the particular classification algorithm. In detail, the number of matrix elements created by the libsvm training function, which are necessary to run the evaluated SVM model, were used for evaluating the cardinality of SVM parameters. Particularly they were: *rho*, *sv_coef*, and *SVs* [30]*.* The values of sample rate were: 5 Hz, 10 Hz, 20 Hz, 40 Hz, and 100 Hz (corresponding to 200, 100, 50, 25 and 10 downsampling step). Again, the statistical analysis has been performed through a Wilcoxon Signed-Rank test with significance threshold set to 0.05. Lastly a combined index, called EOF (Embedding Optimization Factor), that takes into account both performance and computational burden has been calculated. It is defined as

16 $$ \left\{\begin{array}{c}\hfill \mathrm{if}\;\left( N\varTheta > n\theta \right)\to P=\frac{\left( N\varTheta - n\theta \right)}{N\varTheta}\cdot 100\hfill \\ {}\hfill \mathrm{if}\;\left( N\varTheta \le n\theta \right)\to P=0\kern3.839998em \hfill \\ {}\hfill EOF=\frac{2\cdot \left(F1 Score\cdot P\right)}{\left(F1 Score+P\right)},\kern3.119999em \hfill \end{array}\right. $$

where NΘ is the maximum acceptable number of parameters. This index plays a paramount role in the implementation of these algorithms in embedded systems, where memory storage and program memory are limited. To this purpose, as representative example, NΘ has been chosen as equal to the maximum number of parameters storable into a 256 KB memory, which is typically used for high performance embedded microcontrollers applied to prosthetic hands (e.g. Touch Bionics I-Limb, Ultra and Robo-Limb). As each parameter is coded as a float which 4 memory bytes are needed to just store one of them, hence, for our example, the maximum number of storable parameters is 64 ∙ 10^3^ classification parameters. This is an application example of how that index and NΘ can be evaluated, but the same method can be applied taking into account different size of memory and/or other constraints, such as the available RAM memory or the evaluation time for a single classification (which is related to the microcontroller clock frequency).

In the third part a comparative analysis among the three non-linear classifiers and the LDA was carried out. Since LDA was trained and tested with data sampled at 1 kHz (without downsampling), NLR, MLP and SVM models with the highest EOF values on GS were taken for the comparison. Again, the analysis was performed taking into account classification performance, computational burden and EOF index. The statistical analysis was performed through a Wilcoxon Signed-Rank test with significance threshold set to 0.05.

## Results

The results are presented in boxplots where the central line represents the median value; the edges of the box are the 25th and the 75th percentiles; the whiskers give the range of the data without outliers; solid markers represent the mean value.

### Max degree of polynomial features for NLR

**Fig. 4:**
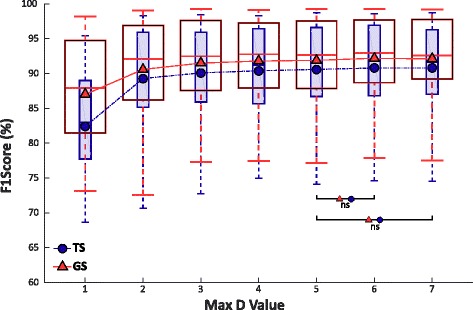
F1Score of Test Set (smaller boxes) and Generalization Set (bigger boxes) of 5 classes over the maximum value of variable D calculated from 30 people with trans-radial amputation. The figure also shows the trend of the mean value for both Sets. Statistical non-significance over value 5 is shown by “ns”

Figure 4 shows the values of F1Score of TS and GS over the max degree of polynomial features (indicated with D) applied as input to NLR.

In both cases, the maximum is reached by setting 7 as maximum D value, but the Wilcoxon Signed-Rank test applied to the F1Score values points out no statistically significant difference for polynomial features over the value 5 for both GS and TS. The result seems to indicate that, for people with trans-radial amputation, the system performance saturates setting the maximum D value of the polynomial features over value 5 as showed in Fig. 3 by the trend lines of the mean values.

### Max number of hidden layers for MLP

**Fig. 5:**
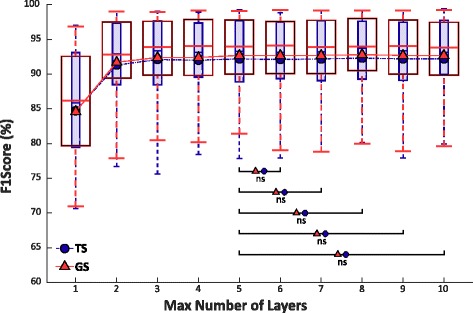
F1Score of Test Set (smaller boxes) and Generalization Set (bigger boxes) of 5 classes over the maximum number of layers having fixed at 30 the maximum number of neurons for each hidden layer calculated from 30 people with trans-radial amputation. The figure also shows the trend of the mean value for both Sets. Statistical non-significance over value 5 is shown by “ns”

Figure 5 shows the values of F1Score of TS and GS over the max number of hidden layers. Each hidden layer has maximum 30 neurons for MLP.

In both cases, the best performance is obtained for a maximum number of layers equal to 8, but the Wilcoxon Signed-Rank test applied to the values of achieved F1Score values points out no statistically significant difference over 5 hidden layers for both GS and TS. This probably means that for people with trans-radial amputation the system performance saturates for a maximum number of hidden layers over the value 5.

### Max number of neurons for MLP

**Fig. 6:**
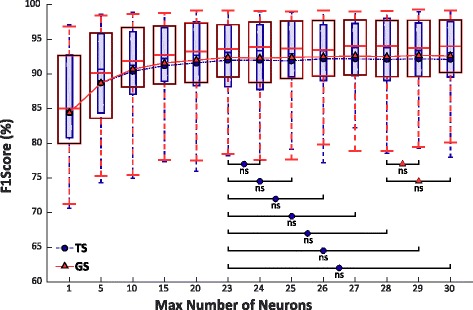
F1Score of Test Set (smaller boxes) and Generalization Set (bigger boxes) of 5 classes over the maximum number of neurons for each layer. The maximum number of hidden layers calculated from 30 people with trans-radial amputation has been fixed at 5. The figure also shows the trend of the mean value for both Sets. Statistical non-significances over value 23 for and overvalue 28 for GS are shown by “ns”

Figure 6 summarizes the values of F1Score of TS and GS with respect to the max number of neurons for a MLP with maximum 5 hidden layers varying by 5 the number of neurons until the value 23, for compactness. The Wilcoxon Signed-Rank test applied to the achieved values of F1Score points out no highly statistically significant difference over 23 for TS and over 28 for GS. This probably means that for people with trans-radial amputation the system performance saturates for a maximum number of neurons between 23 and 28 depending on the frequency of the signals to classify.

### NLR, MLP, SVM comparison based on TR sampling rate

**Fig. 7:**
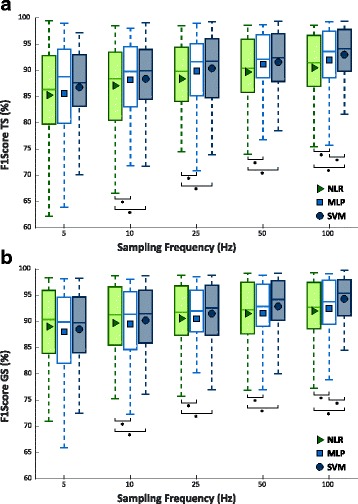
F1Score values from 30 people with trans-radial amputation increasing the sampling frequency of the dataset used to train and cross validate the NLR, MLP, and SVM algorithms and 5 classes. Statistical significance is shown by “*”. **a**) F1Score values for Test Set; **b**) F1Score values for Generalization Set

Figure 7 shows the values of F1Score of TS and GS, obtained training the classifiers on TR sampled at increasing sampling rate (or at decreasing downsampling step) for NLR, MLP, and SVM. As mentioned in Sect. II, NLR and MLP has been optimized by using the results previously obtained by limiting to 5 the maximum D value, for NLR and to 5 and 28 the maximum number of layers and neurons, respectively, for MLP. Afterwards, performance of NLR, MLP, and SVM were compared, at different sampling frequencies of the dataset used to train the algorithms, through a Wilcoxon Signed-Rank test. For both TS and GS the analysis reports no statistically difference between the three classifiers when training the algorithms with a 5 Hz sampled dataset, and that NLR achieved significant lower value than MLP and SVM with the others sampling frequencies. Conversely, MLP achieved statistically significant lower performance than SVM only using a 100 Hz frequency.

### NLR, MLP, SVM comparison based on computational burden

**Fig. 8:**
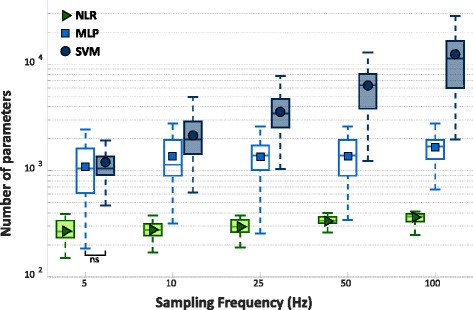
Number of classification parameters from 30 people with trans-radial amputation increasing the sampling frequency of the dataset used to train and cross validate the NLR, MLP, and SVM algorithms and 5 classes. The y ax is in logarithmic scale. Statistical non-significance is shown by “ns”

Figure 8 shows the number of classification parameters (nθ), obtained training the classifiers on datasets sampled at increasing sampling rate (or at decreasing downsampling step) for NLR, MLP, and SVM. Variable nθ is regarded as an index quantifying the algorithm computational burden. Again NLR and MLP has been optimized thanks to the previously obtained results. As the model of the classifier adopted for TS and GS is the same, also the complexity in the two cases is the same.

By comparing the algorithms at different sampling rates for the dataset used to train the three algorithms, it can be observed that SVM is always characherized by the highest computational cost, while NLR by the lowest one. While NLR and MLP remain statistically different they retained values of nθ that always belong to the same order of magnitude (10^2^ for NLR and 10^3^ for MLP), SVM initially scores values statistically equals to MLP (5 Hz) and then diverged with respect to the sampling rate. This difference in behavior of the SVM classifier is due to its unique achitecture that generates a number of landmarks (6), which are strictly related to the number of the classification parameters, depending on the numerosity of the dataset used to train the algorithm. Therefore, the higher the sampling frequency the more numerous the TR will be and, consequently, a high number of landmarks to represent the data is needed. All the others comparisons proved to be statistically different among them.

### NLR, MLP, SVM comparison based on EOF

**Fig. 9:**
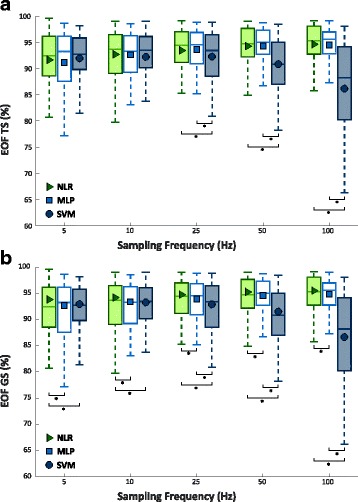
EOF values from 30 people with trans-radial amputation increasing the sampling frequency of the dataset with 5 classes used to train and cross validate the NLR, MLP, and SVM algorithms. Statistical significance is shown by “*”. a) EOF values for Test Set; b) EOF values for Generalization Set

As previously mentioned in this section it was reported a result of an applicative example comparing NLR, MLP and SVM classifiers using EOF as comparison index. The only constraint adopted in this analysis is the burden on a 256 KB memory that the classification parameters to be stored produce. Figure 9 shows values of EOF for TS and GS, obtained training the classifiers on datasets sampled at increasing sampling rate (or at decreasing downsampling step) for NLR, MLP, and SVM. Again, NLR and MLP were optimized using the results previously obtained. Hence, a comparative analysis among NLR, MLP, and SVM was carried out (first for TS, then for GS).

Except that for TS at 5 Hz sampling frequency (where SVM has obtained the maximum value of EOF) among the three classifiers NLR attained the maximum EOF value for both TS and GS and perhaps, the result means that for people with trans-radial amputation NLR and MLP classifiers represent the best compromise between classification performance and computational burden. The result is even more valuable considering the trend of the value of EOF increasing the sampling rate. In fact, for the NLR and the MLP classifier the value of this index tends to slightly increase, while for the SVM classifier it decreases more and more.

### NLR, MLP, SVM and LDA comparison

**Fig. 10:**
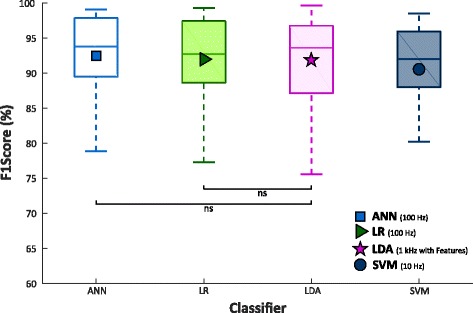
F1Score values from 30 people with trans-radial amputation for MLP, NLR, SVM, tested on GS, and LDA with 5 time domain features, on a 5 classes dataset. NLR and MLP where trained using data sampled at 100 Hz, while SVM using data sampled at 10 Hz. Statistical non-significance is shown by “ns”

In this section the results of the comparative analysis of LDA withNLR, MLP, and SVM classifiers are reported. For comparative purposes, NLR, MLP, and SVM models that obtained the highest EOF values on GS were used. The LDA classifier was considered as ground truth, in terms of performance, number of parameters and EOF index. Figure 10 shows the values of F1Score of GS for NLR, and MLP on TR sampled at 100 Hz and SVM, on TR sampled at 25 Hz, and of TS_30%_ for LDA on TR_70%_ sampled at 1 kHz. By exploiting the previously obtained optimization results, D value was limited to 5 for NLR, while the maximum number of layers and neurons was limited to5 and 28 for MLP. Table 2 shows the numeric values of F1Scores averaged over 30 subjects with trans-radial amputation and the corresponding standard deviation (s) for all the four algorithms.

**Table 2:** Classification performance and computational burden for NLR, MLP and SVM models with highest EOF value on GS and LDA sampled at 1 kHz with features

Classification Algorithm	F1Score	Number of Classification Parameters	EOF
NLR (100 Hz)	92.0 (6.1 s)	362 (41 s)	**95.5 (3.4 s)**
MLP(100 Hz)	**92.5 (5.9 s)**	1654 (605 s)	94.8 (3.2 s)
SVM (10 Hz)	89.5 (7.3 s)	1361 (648 s)	93.3 (4.4 s)
LDA (1 kHz with features)	91.9 (6.5 s)	**155**	**95.5 (3.7 s)**

**Fig. 11:**
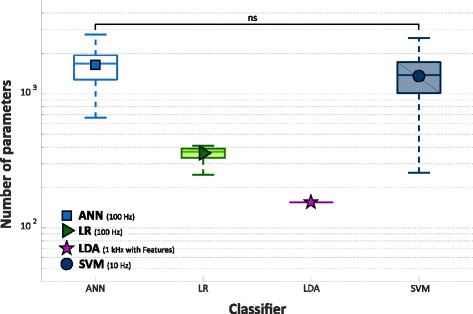
Number of classification parameters from 30 people with trans-radial amputation for MLP, NLR, SVM, and LDA with 5 time domain features, on a 5 classes dataset. NLR and MLP where trained using data sampled at 100 Hz, while SVM using data sampled at 10 Hz. The y ax is in logarithmic scale. Statistical non-significance is shown by “ns”

**Fig. 12:**
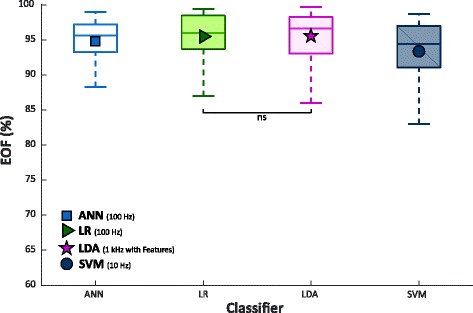
EOF values from 30 people with trans-radial amputation for MLP, NLR, SVM, tested on GS, and LDA with 5 time domain features, on a 5 classes dataset. NLR and MLP where trained using data sampled at 100 Hz, while SVM using data sampled at 10 Hz. Statistical non-significance is shown by “ns”

Mean values and standard deviation of F1Score values, classification parameters and EOF values from 30 people with trans-radial amputation for each classifier involved in this study on a 5 classes dataset. The EOF and F1Score highest values and the lowest number of parameters are highlighted in bold. See Figs. 10-11-12 for a graphic display and statistical significance

A Wilcoxon Signed-Rank test was adopted for the statistica analysis of comparison between NLR, MLP, and SVM and LDA.. The analysis reports no statistically significant difference between LDA and both NLR and MLP classifiers, while SVM achieved significantly lower value than the others. Figure 11 displays the number of classification parameters (nθ and n nβ). Table 2 shows the number of classification parameters averaged over 30 subjects with trans-radial amputation and the corresponding standard deviation (σ) for the four algorithms. The analysis showed that LDA obtained the minimum number of parameters, and no statistically significant difference was observed only between MLP and SVM.

Finally, the EOF index for LDA was evaluated and compared with NLR, MLP and SVM, as showed in Fig. 12 and Table 2. While SVM achieved significantly lower value than the other classifiers, MLP, NLR and LDA showed similar EOF score. The Wilcoxon Signed-Rank showed no statistically significant difference only between the NLR and LDA classifier.

## Discussion

In this study an in-depth analysis has been carried out of three of the most adopted classifiers for EMG signals, i.e. NLR, MLP, and SVM using LDA with time domain feature extraction as ground truth for the final validation of the performed analysis. The choice fell on these because of the extensive discussion in the literature and because of the high performance notwithstanding the extremely different number of classification parameters. In particular, an intensive analysis on data acquired from 30 people with trans-radial amputation was conducted and performance were assessed, with special attention to the problem of developing embedded classifier solutions. Although the type and number of recruited subjects was not sufficient to generalize the results to all kinds of trans-radial amputations, this study wants to provide a solid basis for reflecting upon the trade-off between performance and computational burden of these classifiers.

Six commercial sEMG sensors produced analog signals that were sampled at 1 kHz and used as “raw” input features of the classifiers. In order to speed up the training and the cross validation of NLR, MLP and SVM classification algorithms, downsampling was applied to the data creating one downsampled dataset (TR, CV, and TS) and one dataset containing all the remaining data (GS). While the TR and CV were used to train and cross validate, TS and GS have been used to test the performance of the classifiers.

The performance of NLR and MLP algorithms were firstly evaluated and then analyzed with the Wilcoxon Signed-Rank test for both TS and GS. The results showed that for NLR no significant improvement of performance can be obtained for a degree of polynomial features greater than 5 and that for MLP no significant improvements can be achieved by increasing the complexity of the network up to 5 layers and 23 neurons for TS and 28 neurons for GS, respectively (Fig. 5). This result is very important because sets a boundary on the complexity of the classifier, allowing to reduce the training and cross-validating times when applying these algorithms on raw sEMG data recorded from people with trans-radial amputation. Furthermore, it is also relevant to observe that NLR in the linear case analysis (polynomial features of grade 1) obtained the lowest F1Score value with respect of the other higher grade of polynomial features, suggesting the use of a non-linear classifier when as input features the raw outputs of the Ottobock sEMG sensors are used.

After this preliminary investigation, a comparative analysis among the NLR, MLP, and SVM algorithms was performed using data at different frequencies (5 Hz, 10 Hz, 20 Hz, 40 Hz, and 100 Hz) as TR, CV and TS. The comparison pointed out that the sampling rate and the classification performance increased at the same time (Fig. 7). In fact, for all the algorithms the maximum performance was obtained with 100 Hz sampling rate, however, increasing the sampling rate also tends to elevate the number of classification parameters, used as index of computational burden of the classifier. The analysis showed that, for both classification performance and number of classification parameters (Fig. 8), SVM attains the highest values followed by MLP, and then by NLR. Although downsampling causes a loss of information, classification performance was still high (ranging from 91.1% to 94.5%) meaning that the signals kept the main content related to the gesture. The reason is that, for constructing a decision boundary, it is not necessary to use high frequency sampled data during the classifier training phase; data with similar range, dispersion and redundancy are required. This also explains why GS systematically reports higher performance value than TS. GS contains a larger number of data than TS and, consequently, leads to higher performance scores. Hence, the results carried out from it might better represents the real behavior of the classifiers when data sampled up to 1 kHz are provided as input.

Although when implementing these algorithms on PC systems it is reasonable to choose the one with the highest classification performance, when moving to embedded systems for prosthetic devices, the computational burden is no longer negligible. Hence, in order to investigate the best compromise between performance and computational burden, the EOF index was presented. Using as unique constraint the memory usage, the EOF has been evaluated referring to a standard microcontroller 256 KB memory at different frequencies of TR, CV and TS. As previously reported, this is just an application example but the same method can be applied taking into account different memory values and/or other constraints, such as the available RAM memory and/or the evaluation time for a single classification for any microcontroller. The analysis performed showed that, for people with trans-radial amputation and using sampled sEMG signals to more than 5 Hz as input, the algorithm that produces the best compromise is NLR, with the highest values of EOF (95.5%), closely followed by MLP (94.8%). Conversely, SVM algorithm, which obtained the highest classification performance, presents considerably lower values of EOF (93.3%) than the other two algorithms (Fig. 9); this means that high performance is achieved at the expenses of a sharp increase of the computational burden and memory usage. Hence, it is possible to summarize that in order to choose the most suitable classifier in a real application with data sampled at the same frequency used for train and cross validate the algorithm, there is no difference between NLR, MLP, and SVM up to 10 Hz, while from 10 to 100 Hz SVM becomes significantly disadvantageous with respect to the other two classifiers, which did not show significant difference. On the other hand, for use in a real application with data sampled at higher frequency (up to 1 kHz) than the ones used to train and cross validate the algorithms, NLR resulted to be the most suitable clearly representing the best compromise between classification performance and computational burden. Furthermore, the analysis suggests, among the tested cases, a downsampling step equal to 10 (100 Hz) for the training and the cross validation of NLR and MLP algorithms, and equal to 100 (10 Hz) for SVM.

Finally, a comparison between each of the three non-linear classifiers and LDA was carried out. Since LDA was trained and tested with data sampled at 1 kHz (without downsampling), NLR, MLP and SVM models with the highest EOF values on GS for performance, number of parameters and EOF index were used for the comparative analysis. This analysis pointed out no statistically significant difference between NLR and LDA in terms of performance and EOF index (Figs. 10-11-12, Table 2) confirming the results of the previously showed comparisons (Figs. 7-8-9) despite LDA reported the minimum computational burden. Therefore, this result is also more appreciable if we consider that NLR was trained and tested using raw sEMG data. So, this study shows that it is possible to use non-linear classification algorithms on raw sEMG signals recorded from people with trans-radial amputation also for embedded applications. Furthermore, since LDA and NLR retained statistically similar value for both performance and computational burden, it is possible to speculate that the features extraction step linearize the classification problem at the expense of a delay on the class evaluation time and on the readiness of the system during the transition between two different gestures. Indeed, using raw sEMG signals as input features the class evaluation time and system readiness approximate the sampling time; on the other hand, using features based on time windowing, the class evaluation time equals the window shift and the readiness delay is around the half of the time window length.

It is worth noticing that, when transient EMG signals are included in classifier training, system controllability and performance are shown to improve [31]; conversely, offline classification accuracy degrades. This comparative study was grounded on steady state sEMG signals, however, this does not affect our comparative analysis, since the experimental data were the same for all the analysed classifiers.

## Conclusions

In this study the NLR, MLP and SVM classification algorithms were developed, tested and optimized on a dataset of 5 hand gestures classes composed of the data recorded from 30 people with trans-radial amputation, using 6 commercial sEMG sensors. After evaluating the maximum complexity of the NLR and MLP algorithms needed to apply pattern recognition on this population, the comparative analysis among the three algorithms was carried out. It pointed out that, for both classification performance and number of classification parameters, SVM attains the highest values followed by MLP, and then by NLR. Hence, in order to investigate the best compromise between performance and computational burden, the EOF index was presented. The analysis performed showed that, for people with trans-radial amputation and using sampled sEMG signals to more than 5 Hz as input, the algorithm that reached the best compromise is NLR (with the highest value of EOF) closely followed by MLP. This result was also confirmed by the comparative analysis with LDA with time domain features, which showed no statistically significant difference with NLR. The proposed analysis would provide innovative engineering tools and indications on how to choose the most suitable classifier, and its specific internal settings, based on the application and the desired results for prostheses control. As the research has reached an advanced grade of accuracy, these algorithms were proved and the embedding is necessary for the realization of prosthetic devices. Future developments will exploit the results of this study by extending the analysis to transient EMG signals, and developing a control unit embedding pattern recognition algorithms for people with trans-radial amputation. Then, measures of system robustness and reliability will be carried out and performance of real-time myoelectric pattern recognition control of a multifunctional upper-limb prosthesis will be evaluated by means of specific tests (e.g. TAC test [16]).

## Abbreviations

ANN: Artificial Neural Network
CV: Cross Validation Set
DOFs: Degrees of freedom
GS: Generalization Set
L-BFGS: Limited Memory Broyden-Fletcher-Goldfarb-Shanno
LDA: Linear Discriminant Analysis
LR: Logistic Regression
MLP: Multi-Layer Perceptron
NLR: Non-Linear Logistic Regression
RBF: Radial Basis Function
sEMG: Surface electromyography
SVM: Support Vector Machine
TMR: Targeted Muscled Re-innervation
TR: Training Set
TS: Test Set

## Footnotes

1 note that in the following SVM will be used to indicate SVM with RBF kernel

2 note that in the following LDA will be used to indicate LDA with 5 time domain features
